# Taxifolin Attenuates Remote Lung Injury Induced by Hepatic Ischemia–Reperfusion in Rats

**DOI:** 10.3390/molecules31071134

**Published:** 2026-03-30

**Authors:** Serkan Erbatur, Meral Erdal Erbatur, Fırat Şahin, Hüseyin Bilge, Aysun Ekinci

**Affiliations:** 1 Department of Plastic, Reconstructive and Aesthetic Surgery, Faculty of Medicine, Dicle University, 21280 Diyarbakır, Turkey; 2 Department of Anesthesiology and Reanimation, Faculty of Medicine, Dicle University, 21280 Diyarbakır, Turkey; 3 Division of Infertility, Turan Çetin In-Vitro Fertilization Center, Çukurova, 01360 Adana, Turkey; 4 Division of General Surgery, Gazi Yaşargil Training and Research Hospital, 21010 Diyarbakır, Turkey; huseyin.bilge@sbu.edu.tr; 5 Department of Biochemistry, Faculty of Medicine, Dicle University, 21280 Diyarbakir, Turkey

**Keywords:** hepatic ischemia–reperfusion, taxifolin, remote lung injury, oxidative stress, NF-κB, HMGB1

## Abstract

Background: Hepatic ischemia–reperfusion (I/R) injury induces systemic oxidative stress and inflammatory responses that may lead to remote lung injury. This study investigated whether taxifolin attenuates hepatic I/R-induced lung damage and examined the involvement of the nuclear factor-κB (NF-κB) and high-mobility group box-1 (HMGB1) signaling axis. Methods: Twenty-eight male Wistar rats were divided into four groups (*n* = 7): control, taxifolin, hepatic I/R, and taxifolin+I/R. Serum oxidative stress markers (malondialdehyde [MDA], interleukin [IL]-6, total antioxidant/oxidant status [TAS/TOS]) and wet-to-dry lung weight ratio were measured. Lung tissues were evaluated histopathologically and immunohistochemically for NF-κB and HMGB1 expression. Bioinformatics pathway enrichment and molecular docking analyses were also performed. Results: Hepatic I/R significantly increased serum MDA, IL-6, and TOS levels and decreased TAS (*p* < 0.05). Severe lung injury was observed in the hepatic I/R group (median score: 11), whereas taxifolin pretreatment significantly reduced the injury score (median score: 5, *p* < 0.001). NF-κB and HMGB1 expression were markedly elevated following hepatic I/R and significantly decreased with taxifolin treatment (*p* < 0.05). A strong positive correlation was found between NF-κB and HMGB1 expression (r = 0.82, *p* < 0.001). Pathway enrichment analysis indicated involvement of Toll-like receptor (TLR)-related inflammatory signaling, and docking analysis demonstrated favorable binding of taxifolin to TLR4 and NF-κB p65. Conclusion: Taxifolin attenuated hepatic I/R-induced lung injury by reducing oxidative stress and suppressing HMGB1–TLR4–NF-κB-mediated inflammatory signaling.

## 1. Introduction

Hepatic I/R injury represents a major clinical challenge in several surgical and clinical settings, including liver transplantation, hepatic resection, trauma, and hemorrhagic shock [1,2]. The temporary interruption of hepatic blood flow followed by reperfusion triggers a complex cascade of metabolic, inflammatory, and oxidative events that contribute to substantial tissue damage [3,4]. Although the liver is the primary organ affected by ischemia–reperfusion, accumulating evidence indicates that the resulting systemic inflammatory response can induce injury in distant organs. Among these organs, the lung is particularly vulnerable and often develops acute inflammatory alterations that may progress to severe respiratory complications [5].

The pathophysiology of remote lung injury following hepatic I/R is primarily driven by excessive oxidative stress and inflammatory signaling. During reperfusion, a rapid burst of reactive oxygen species (ROS) is generated, which disrupts cellular redox balance and initiates lipid peroxidation, protein oxidation, and DNA damage. Simultaneously, activated Kupffer cells and infiltrating neutrophils release pro-inflammatory cytokines and damage-associated molecular patterns (DAMPs), amplifying systemic inflammation [6,7]. Key signaling pathways such as nuclear factor-κB [8,9] and HMGB1 play central roles in mediating these processes [10], ultimately contributing to endothelial dysfunction, increased vascular permeability, and inflammatory cell infiltration in pulmonary tissues [11,12,13,14,15,16].

Given the critical role of oxidative stress and inflammation in hepatic I/R-associated lung injury, pharmacological agents with potent antioxidant and anti-inflammatory properties have attracted increasing attention as potential protective strategies [17]. Taxifolin (dihydroquercetin) is a naturally occurring flavonoid widely distributed in several plant species [18]. It has been reported to exhibit strong antioxidant, anti-inflammatory, and cytoprotective effects in various experimental models [19]. Previous studies have demonstrated that taxifolin can attenuate oxidative stress, inhibit inflammatory signaling pathways, and modulate cellular defense mechanisms in renal [20], cardiac [21], hepatic [22], and neural [23] tissues. However, despite these promising biological properties, its potential protective effects against hepatic ischemia–reperfusion-induced remote lung injury remain insufficiently investigated.

Therefore, the present study aimed to evaluate the protective effects of taxifolin against hepatic I/R-induced lung injury using biochemical, histopathological, and immunohistochemical approaches. In addition, bioinformatics analyses and molecular docking were employed to explore the potential molecular interactions and signaling pathways underlying the protective mechanisms of taxifolin. By integrating experimental and computational approaches, this study seeks to provide a more comprehensive understanding of the potential therapeutic role of taxifolin in ischemia–reperfusion-related organ injury.

## 2. Results

### 2.1. Effects of Hepatic I/R and Taxifolin on Oxidative Stress and Inflammatory Markers

Serum oxidative stress and inflammatory parameters are presented in Table 1. Significant differences were observed among the experimental groups for MDA, IL-6, TAS, and TOS levels. The hepatic I/R group exhibited markedly elevated MDA levels compared with the control and taxifolin groups, indicating increased lipid peroxidation following hepatic I/R injury. Pretreatment with taxifolin significantly reduced MDA levels in the taxifolin + I/R group compared with the hepatic I/R group (*p* = 0.004), suggesting attenuation of oxidative membrane damage. Similarly, IL-6 levels were significantly increased in the hepatic I/R group, reflecting a pronounced systemic inflammatory response. Taxifolin pretreatment significantly decreased IL-6 concentrations in the taxifolin + I/R group (*p* = 0.003). Regarding antioxidant status, TAS levels were significantly higher in the taxifolin group compared with the control group (*p* = 0.006), indicating enhanced baseline antioxidant capacity. However, TAS levels did not significantly differ between the hepatic I/R and taxifolin + I/R groups (*p* = 0.096). In contrast, TOS levels were significantly elevated in the hepatic I/R group, demonstrating increased oxidative burden. Taxifolin pretreatment significantly reduced TOS levels compared with the hepatic I/R group (*p* = 0.002), indicating partial restoration of redox balance.

### 2.2. Effect of Taxifolin on Lung Wet-to-Dry Weight Ratio Following Hepatic I/R

The wet-to-dry lung weight was calculated and was shown in Table 2. The ratio was markedly increased in the hepatic I/R group (5.82 ± 0.35) compared with the control (4.48 ± 0.21) and taxifolin (4.37 ± 0.24) groups, indicating significant pulmonary edema. Pretreatment with taxifolin significantly reduced this ratio (4.89 ± 0.27, *p* < 0.05 vs. hepatic I/R); however, the values in the taxifolin + I/R group remained slightly higher than those of the control group (*p* < 0.05), supporting the conclusion that lung injury was ameliorated but not completely normalized.

### 2.3. Histopathological Lung Injury

**Figure 1:**
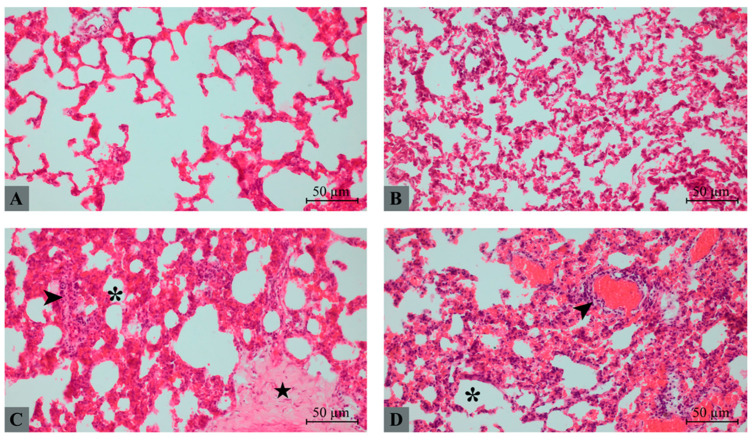
Representative H&E stained sections of lung tissues from experimental groups: (**A**) Control, (**B**) taxifolin, (**C**) hepatic I/R, and (**D**) taxifolin + I/R. Arrowhead: inflammatory infiltration, asterisk: intra-alveolar space, star: fibrosis. Scale bar: 50 µm.

Representative H&E-stained lung sections are shown in Figure 1. In the control group, lung architecture was preserved with thin interalveolar septa and open alveolar spaces. No evidence of inflammatory infiltration, hemorrhage, or edema was observed (Figure 1A). The taxifolin group demonstrated largely preserved alveolar structure with only mild septal thickening and minimal cellular infiltration (Figure 1B). In contrast, the hepatic I/R group exhibited severe histopathological alterations, including marked septal thickening, inflammatory cell infiltration within alveolar and interstitial compartments, focal hemorrhage, and partial disruption of alveolar architecture. Taxifolin pretreatment markedly attenuated these pathological alterations (Figure 1C). In the taxifolin + I/R group, alveolar spaces were relatively preserved, and the severity of inflammatory infiltration, hemorrhage, and septal thickening was significantly reduced compared with the hepatic I/R group (Figure 1D).

Quantitative histopathological scoring revealed significant differences among groups (Table 3). Lung histopathological injury scores differed significantly among the groups (*p* < 0.001). The hepatic I/R group demonstrated significantly higher scores for alveolar edema, hemorrhage, leukocyte infiltration, and septal thickening compared to the control and taxifolin groups (*p* < 0.05 for all comparisons). In the taxifolin + I/R group, all histopathological parameters were significantly reduced compared to the hepatic I/R group (*p* < 0.05). The total lung injury score was 11 (10–13) in the hepatic I/R group, whereas it decreased to 5 (4–7) in the taxifolin + I/R group.

### 2.4. NF-κB Immunohistochemical Expression

**Figure 2:**
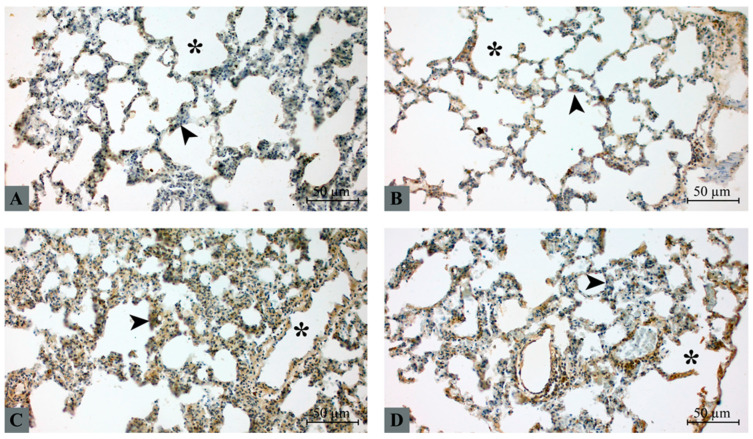
Immunohistochemical detection of NF-κB expression in lung tissue. Representative lung sections from (**A**) control, (**B**) taxifolin, (**C**) hepatic I/R, and (**D**) taxifolin + I/R groups. Brown DAB staining indicates NF-κB immunoreactivity. Arrowhead: alveolar epithelium, asterisk: intra-alveolar space. Scale bar: 50 µm.

NF-κB immunoreactivity was observed predominantly in alveolar epithelial cells and interalveolar septal cells (Figure 2). In the control group, NF-κB expression was minimal and mainly restricted to weak cytoplasmic staining in septal cells (Figure 2A). Similarly, the taxifolin group showed only mild cytoplasmic staining with limited distribution (Figure 2B). In contrast, the hepatic I/R group demonstrated markedly increased NF-κB expression, characterized by strong cytoplasmic staining and focal nuclear localization within alveolar septal cells and inflammatory infiltrates (Figure 2C). Taxifolin pretreatment significantly attenuated NF-κB activation. In the taxifolin + I/R group, immunoreactivity was reduced to mild-to-moderate levels with limited nuclear localization compared with the hepatic I/R group (Figure 2D).

### 2.5. HMGB1 Immunohistochemical Expression

**Figure 3:**
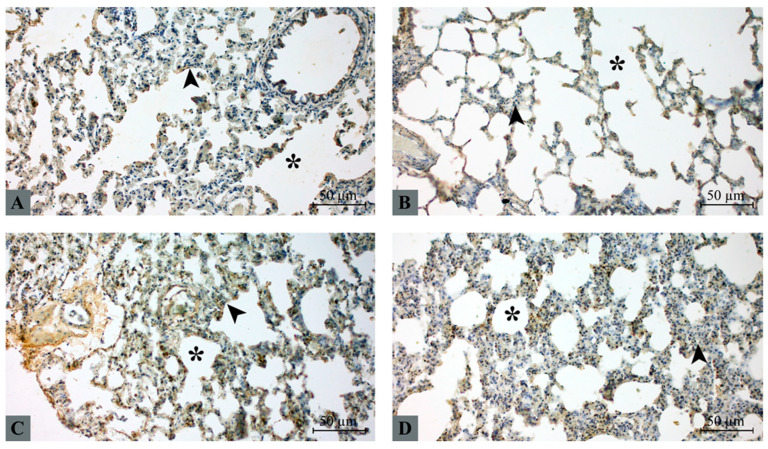
Immunohistochemical detection of HMGB1 expression in lung tissue. Representative lung sections from (**A**) control, (**B**) taxifolin, (**C**) hepatic I/R, and (**D**) taxifolin + I/R groups. Brown DAB staining indicates HMGB1 immunoreactivity. Arrowhead: alveolar epithelium, asterisk: intra-alveolar space. Scale bar: 50 µm.

HMGB1 expression was detected in alveolar epithelial cells and interalveolar septal cells (Figure 3). In the control group, HMGB1 expression was weak and primarily localized within the nucleus, with minimal cytoplasmic staining (Figure 3A). The taxifolin group demonstrated a similar staining pattern with mild nuclear expression and limited cytoplasmic distribution (Figure 3B). In contrast, the hepatic I/R group exhibited markedly increased HMGB1 expression, characterized by prominent cytoplasmic staining and focal extracellular deposition, indicating translocation of HMGB1 from the nucleus to the cytoplasm (Figure 3C). Taxifolin pretreatment attenuated these changes. In the taxifolin + I/R group, HMGB1 staining intensity was reduced, with partial restoration of the nuclear localization pattern and decreased cytoplasmic accumulation (Figure 3D).

### 2.6. Correlation Analysis Between NF-κB and HMGB1

Significant differences were observed among the groups in terms of NF-κB and HMGB1 immunohistochemical expression scores (*p* < 0.001) (Table 4). Both NF-κB and HMGB1 expression scores were significantly higher in the hepatic I/R group compared to the control and taxifolin groups (*p* < 0.05). In the taxifolin + I/R group, expression scores of both markers were significantly reduced compared to the hepatic I/R group (*p* < 0.05).

A strong and statistically significant positive correlation was identified between NF-κB and HMGB1 immunohistochemical expression scores (Spearman r = 0.82, *p* < 0.001) (Table 5). This finding indicates that inflammatory activation and DAMP-mediated signaling increased concurrently in lung injury secondary to hepatic ischemia–reperfusion.

### 2.7. Reactome Pathway Enrichment Analysis of the HMGB1–NF-κB Interaction Network

**Figure 4:**
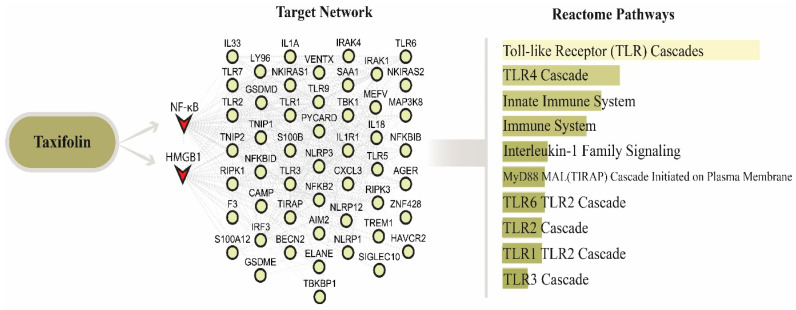
Protein–protein interaction network and pathway enrichment analysis of the HMGB1–NF-κB axis.

The PPI network constructed for HMGB1 and NF-κB consisted of 52 nodes and 488 edges. Reactome pathway enrichment analysis performed using this PPI network demonstrated that the following pathways were significantly associated with the network: TLR Cascades (*p* = 4.529 × 10^−47^), TLR4 Cascade (*p* = 4.843 × 10^−36^), Innate Immune System (*p* = 1.382 × 10^−34^), Immune System (*p* = 2.116 × 10^−33^), IL-1 Family Signaling (*p* = 2.027 × 10^−30^), MyD88 MAL (TIRAP) Cascade Initiated on Plasma Membrane (*p* = 3.717 × 10^−30^), TLR6 TLR2 Cascade (*p* = 3.717 × 10^−30^), TLR2 Cascade (*p* = 6.296 × 10^−30^), TLR1 TLR2 Cascade (*p* = 6.296 × 10^−30^), and TLR3 Cascade (*p* = 8.776 × 10^−29^). Collectively, these results indicate that TLR signaling pathways and immune system-related pathways were prominently represented within the HMGB1–NF-κB interaction network (Figure 4).

### 2.8. Molecular Docking Analysis

**Figure 5:**
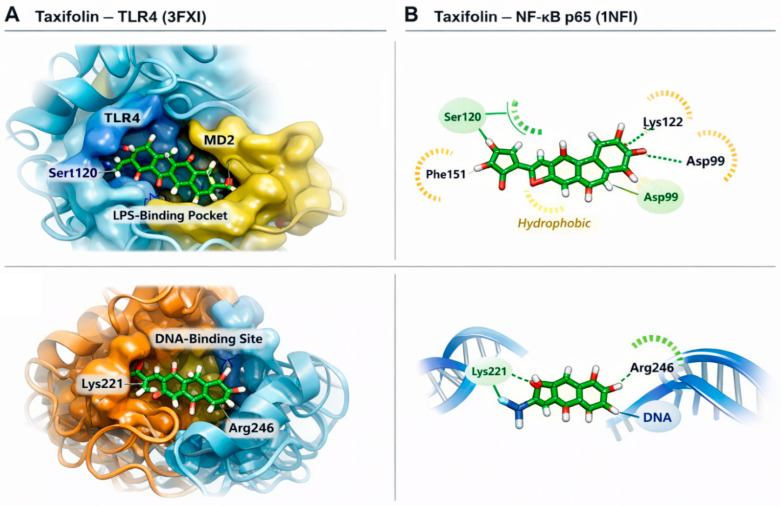
Molecular docking analysis of taxifolin with TLR4 and NF-κB p65. (**A**) Three-dimensional docking pose of taxifolin within the TLR4–MD2 complex (PDB ID: 3FXI). Taxifolin is positioned inside the LPS-binding pocket at the TLR4–MD2 interface. Key interacting residues including Ser120, Lys122, Asp99, and Phe151 are indicated. Hydrogen bonds are represented as dashed lines. (**B**) Docking pose of taxifolin within the DNA-binding domain of NF-κB p65 (PDB ID: 1NFI). Taxifolin interacts with critical DNA-contact residues Lys221 and Arg246. The spatial arrangement suggests potential steric interference with DNA binding.

Taxifolin demonstrated favorable binding affinity toward TLR4 and NF-κB p65 proteins (Figure 5). The strongest interaction was observed with TLR4 (−8.4 kcal/mol), followed by NF-κB p65 (−7.2 kcal/mol). Within the TLR4–MD2 complex, taxifolin was positioned inside the LPS-binding pocket and formed hydrogen bonds with Ser120, Lys122, and Asp99. Additional hydrophobic interactions with Phe151 and Ile124 contributed to stabilization within the receptor cavity. Docking analysis of NF-κB p65 revealed that taxifolin localized within the DNA-binding domain, interacting with Lys221 and Arg246. This positioning suggests potential steric interference with DNA recognition, thereby attenuating transcriptional activation. Collectively, these in silico findings indicate that taxifolin may directly target both upstream (TLR4) and downstream (NF-κB p65) components of the HMGB1–TLR4–NF-κB inflammatory axis.

## 3. Discussion

Hepatic I/R injury is known to trigger systemic inflammatory responses that may extend beyond the liver and cause secondary damage in distant organs, particularly the lungs [24,25]. In the present study, we investigated whether taxifolin could attenuate remote lung injury induced by hepatic I/R through modulation of oxidative stress and inflammatory signaling pathways. Our findings demonstrate that hepatic I/R markedly increased oxidative stress markers, promoted histopathological lung injury, and enhanced NF-κB and HMGB1 expression in lung tissue, whereas taxifolin pretreatment significantly mitigated these alterations, consistent with experimental models of remote lung injury following hepatic insult by Eltzschig et al. [26] and Nastos et al. [27].

The biochemical findings of the present study indicate that hepatic I/R resulted in a pronounced oxidative imbalance, as evidenced by elevated serum MDA and TOS levels together with increased IL-6 concentrations. Lipid peroxidation products such as MDA are widely recognized indicators of oxidative membrane damage, and their elevation reflects enhanced reactive oxygen species (ROS) production during reperfusion injury [28,29]. Consistent with previous studies of Li et al. [30] and Topal et al. [31], hepatic I/R markedly increased systemic oxidative stress and inflammatory cytokine release, both of which contribute to remote organ damage. Importantly, taxifolin pretreatment significantly reduced MDA and TOS levels while suppressing IL-6 elevation, suggesting that taxifolin exerts potent antioxidant and anti-inflammatory effects under ischemia–reperfusion conditions.

These biochemical alterations were paralleled by significant histopathological changes in lung tissue. Animals subjected to hepatic I/R exhibited characteristic features of acute lung injury, including alveolar septal thickening, inflammatory cell infiltration, hemorrhage, and disruption of normal alveolar architecture. Such structural changes are consistent with the well-established concept that hepatic I/R induces remote pulmonary damage through systemic inflammatory mediators and circulating oxidative molecules. In contrast, taxifolin pretreatment markedly alleviated these histopathological alterations, indicating that the compound may protect pulmonary tissue integrity during systemic inflammatory stress.

At the molecular level, our immunohistochemical findings demonstrated that hepatic I/R markedly increased NF-κB activation in lung tissue. NF-κB is a key transcription factor regulating inflammatory gene expression and is known to be activated during oxidative stress and ischemia–reperfusion injury [32]. Increased NF-κB activation has been associated with the transcription of multiple pro-inflammatory mediators that contribute to tissue injury and inflammatory cell recruitment [33]. In the present study, taxifolin pretreatment significantly attenuated NF-κB immunoreactivity, consistent with experimental data demonstrating that taxifolin modulates NF-κB signaling in ischemia-related models [22,34,35]. The findings of this study supports the hypothesis that taxifolin may suppress inflammatory signaling pathways activated during hepatic I/R.

Similarly, HMGB1 expression was markedly elevated in lung tissues following hepatic I/R injury. HMGB1 is a damage-associated molecular pattern (DAMP) molecule that is released from stressed or necrotic cells and acts as a potent mediator of sterile inflammation [36,37]. Extracellular HMGB1 has been shown to activate innate immune responses through receptors such as Toll-like receptor 4 (TLR4), ultimately leading to downstream activation of NF-κB signaling pathways [38]. In agreement with this mechanism, we observed a strong positive correlation between HMGB1 and NF-κB expression scores, suggesting coordinated activation of inflammatory signaling cascades during hepatic I/R-induced lung injury.

Taxifolin pretreatment significantly reduced HMGB1 immunoreactivity in lung tissue, indicating that the compound may interfere with DAMP-mediated inflammatory signaling. This observation is consistent with previous studies demonstrating that flavonoid compounds can suppress HMGB1 release and inhibit downstream inflammatory pathways [38,39]. By reducing HMGB1 signaling and NF-κB activation, taxifolin may attenuate the propagation of systemic inflammatory responses following hepatic ischemia–reperfusion.

To further explore potential molecular mechanisms underlying these observations, we performed a bioinformatics pathway enrichment analysis based on a protein–protein interaction network centered on HMGB1 and NF-κB. The enrichment results suggested that several inflammatory and immune-related pathways, including TLR signaling and NF-κB regulatory pathways, may be involved in the observed responses [25,40]. These findings support the experimental data and provide additional evidence that HMGB1-mediated signaling pathways may play a central role in the development of remote lung injury following hepatic I/R [26]. In addition, molecular docking analysis demonstrated that taxifolin exhibited favorable binding interactions with both TLR4 and NF-κB p65 proteins. These computational findings suggest that taxifolin may directly interact with key components of inflammatory signaling pathways.

However, several limitations should be considered when interpreting the results of this study. First, the study was conducted in an experimental animal model, and therefore the findings may not be directly extrapolated to clinical settings. Second, although the bioinformatics and molecular docking analyses provide mechanistic insights, additional molecular studies (Western blot, gene knockout, etc.) would be required to confirm the direct interaction between taxifolin and the investigated signaling pathways. Third, pulmonary functional parameters were not evaluated. Fourth, findings are based on an acute experimental model, and longer-term outcomes were not assessed. Another limitation of the present study is the absence of a reference drug for comparison, and the use of a single dose of taxifolin (50 mg/kg) without evaluation of a dose–response relationship. Finally, only male rats were included in the experimental design. Sex-related differences in inflammatory and oxidative responses may influence ischemia–reperfusion injury.

Despite these limitations, the present study provides integrated biochemical, histopathological, immunohistochemical, and computational evidence supporting the protective role of taxifolin in hepatic I/R-induced remote lung injury. To our knowledge, this is one of the few experimental studies evaluating the protective effects of taxifolin specifically in remote lung injury secondary to hepatic ischemia–reperfusion while simultaneously exploring the HMGB1–TLR4–NF-κB inflammatory signaling axis through combined biochemical, histopathological, immunohistochemical, and in silico approaches.

## 4. Materials and Methods

### 4.1. Animals and Ethical Approval

All experimental procedures were conducted in accordance with the Guide for the Care and Use of Laboratory Animals and complied with the ARRIVE guidelines. The study protocol was approved by the Dicle University, Experimental Animals Local Ethics Committee (Approval No: E-35582840-020-476060). Twenty-eight adult male Wistar albino rats weighing 250–300 g were used in the study. Rats were housed under controlled environmental conditions (22 ± 2 °C temperature, 50–60% humidity, and a 12 h light/dark cycle). Standard laboratory chow and water were provided ad libitum. All animals were allowed to acclimatize to the laboratory conditions for one week before the experimental procedures.

### 4.2. Experimental Design

After the acclimatization period, the rats were randomly divided into four experimental groups (*n* = 7 per group):

1. Control group: Rats underwent sham surgery consisting of anesthesia, midline laparotomy, and exposure of the hepatic pedicle without vascular occlusion. Animals received the same volume of vehicle solution (0.9% saline) without taxifolin administration.
2. Taxifolin group: Rats received taxifolin (50 mg/kg/day, orally) for 21 consecutive days without induction of hepatic I/R injury.
3. Hepatic I/R group: Rats were subjected to hepatic ischemia followed by reperfusion without taxifolin treatment and received the same volume of vehicle solution (0.9% saline).
4. Taxifolin + I/R group: Rats received taxifolin (50 mg/kg/day, orally) for 21 consecutive days prior to the induction of hepatic I/R injury.

### 4.3. Taxifolin Administration

Taxifolin (CAS No. 480-18-2; purity ≥ 98%, Sigma-Aldrich, St. Louis, MO, USA) was used in this study and dissolved in 0.9% saline immediately before administration. The selected dose (50 mg/kg) was based on previous experimental studies demonstrating that this dose represents an effective biologically active dose of taxifolin in rodent models [41,42,43]. Animals in the control and I/R groups received the same volume of vehicle solution to ensure consistency among groups.

### 4.4. Induction of Hepatic Ischemia–Reperfusion

Hepatic I/R injury was induced according to previously described experimental procedures by Bilge et al. [44]. Rats were anesthetized with ketamine (90 mg/kg) and xylazine (10 mg/kg) administered intraperitoneally. Following midline laparotomy, the hepatic pedicle supplying the left and median liver lobes was occluded using an atraumatic microvascular clamp to induce partial hepatic ischemia. The ischemic period was maintained for 30 min. After the ischemic period, the clamp was removed to allow reperfusion for 2 h. During the procedure, body temperature was maintained using a heating pad.

### 4.5. Sample Collection and Euthanasia

At the end of the reperfusion period, animals were deeply anesthetized with ketamine (90 mg/kg; Ketalar^®^, Pfizer, Istanbul, Turkey) and xylazine (10 mg/kg; Rompun^®^, Bayer, Leverkusen, Germany). Adequate anesthesia was confirmed prior to tissue sampling. Euthanasia was then performed by exsanguination under deep anesthesia to ensure that animals did not experience pain or distress. At the end of the experimental protocol, blood and lung tissue samples were collected. A portion of the lung tissue was immediately weighed to determine the wet weight and subsequently dried in an oven at 60 °C for 48 h for wet-to-dry ratio determination. Separate lung tissue samples were fixed in 10% neutral buffered formalin and used for histopathological and immunohistochemical analyses.

### 4.6. Biochemical Analysis

Following euthanasia, blood samples were centrifuged at 3000 rpm for 8 min to obtain serum. The separated serum samples were stored at −70 °C until biochemical analyses were performed. Biochemical analyses were performed using an automated chemistry analyzer (AU5800; Beckman Coulter Inc., Brea, CA, USA). Absorbance measurements were obtained spectrophotometrically at a wavelength of 532 nm, according to the assay protocols provided by the reagent manufacturers.

#### 4.6.1. Measurement of MDA

Serum MDA levels were measured as an indicator of lipid peroxidation using the modified thiobarbituric acid (TBA) method described by Ohkawa et al. [45]. The principle of the assay is based on the reaction of MDA with TBA under acidic conditions and high temperature, forming a pink-colored MDA–TBA complex. The absorbance of the resulting chromogen was measured spectrophotometrically, and the results were expressed as pg/mL.

#### 4.6.2. Measurement of IL-6

Serum IL-6 levels were determined using a commercial ELISA kit (Cat. No. BLS-1158Ra, BostonChem Inc., Cambridge, MA, USA) according to the manufacturer’s instructions. The assay is based on the quantitative sandwich enzyme immunoassay technique. Results were expressed as pg/mL [46].

#### 4.6.3. Measurement of TAS and TOS

Serum TAS and TOS were measured using commercially available kits (catalog no: RL0017, catalog no: RL0024, Rel Assay Diagnostics, Gaziantep, Turkey, respectively) based on the automated colorimetric methods developed by Ermiş [47]. In this method, TAS reflects the cumulative antioxidant capacity of the sample, whereas TOS represents the total level of oxidant molecules present in the serum. TAS values were expressed as mmol Trolox equivalent/L, while TOS values were expressed as μmol H_2_O_2_ equivalent/L.

### 4.7. Lung Wet-to-Dry Weight Ratio Measurement

Pulmonary edema was quantitatively assessed by determining the lung wet-to-dry weight ratio based on the method described by Matute-Bello et al. [12]. Immediately after sacrifice, lung tissues were excised and gently blotted to remove surface blood. The wet weight of each sample was measured using a precision analytical balance. Subsequently, the tissues were dried in an oven at 60 °C for 48 h until a constant weight was achieved. The dry weight was then recorded, and the wet-to-dry ratio was calculated by dividing the wet weight by the dry weight. This parameter was used as an indicator of pulmonary edema severity.

### 4.8. Histopathological Examination

Excised lung tissues were fixed in 10% neutral buffered formalin and processed using standard histological procedures. Paraffin-embedded tissues were sectioned at 4–5 μm thickness using a rotary microtome and stained with hematoxylin and eosin (H&E) for histopathological examination. Histopathological evaluation was performed under light microscopy according to previously established criteria for experimental acute lung injury described by Matute-Bello et al. [12]. The following morphological parameters were assessed: alveolar edema, alveolar hemorrhage, leukocyte infiltration, and alveolar septal thickening. Each parameter was graded on a semi-quantitative scale ranging from 0 to 4 according to the severity of tissue injury:

0 = no or minimal injury,

1 = mild involvement (<25%),

2 = moderate involvement (25–50%),

3 = severe involvement (50–75%),

4 = very severe involvement (>75%).

The total lung injury score for each specimen was calculated as the sum of the scores obtained for all evaluated parameters. Histopathological evaluations were performed using a light microscope (Imager A2, Carl Zeiss, Oberkochen, Germany) at ×200 magnification. For each sample, at least five randomly selected microscopic fields were examined, and the final score was determined based on the median value of the evaluated fields.

### 4.9. Immunohistochemical Evaluation

Immunohistochemical analysis was performed to determine NF-κB and HMGB1 expression in lung tissue sections using a streptavidin–biotin–peroxidase detection system [48,49]. Formalin-fixed, paraffin-embedded lung tissue blocks were sectioned at 4–5 μm thickness and mounted on poly-L-lysine-coated slides. Sections were deparaffinized in xylene and rehydrated through graded ethanol series to distilled water. Antigen retrieval was performed using ethylenediaminetetraacetic acid (EDTA) buffer solution (pH: 8.0; catalogue no: ab93680, Abcam, Cambridge, MA, USA) in a microwave oven for 15 min. After cooling to room temperature, endogenous peroxidase activity was blocked by incubation with 3% hydrogen peroxide solution (catalogue no: TA-015-HP, Thermo Fisher Scientific, Waltham, MA, USA) for 10 min. To prevent nonspecific binding, sections were incubated with a protein blocking solution (catalogue no: TA-125-UB, Thermo Scientific, Waltham, MA, USA) for 15 min at room temperature. Subsequently, the sections were incubated overnight at 4 °C with primary antibodies against NF-κB (catalogue number: sc-8008, Santa Cruz Biotechnology, Inc., Dallas, TX, USA, dilution ratio: 1/200) and HMGB1 (catalogue number: sc-74085, Santa Cruz Biotechnology, Inc., Dallas, TX, US, USA, dilution ratio: 1/200). Following primary antibody incubation, the sections were washed with phosphate-buffered saline and incubated with a biotinylated secondary antibody (catalog no: TP-125-BN, Thermo Scientific, Waltham, MA, USA) for 30 min at room temperature. A streptavidin–peroxidase complex (catalog no: TS-125-HR, Thermo Scientific, Waltham, MA, USA) was then applied for 30 min. Immunoreactivity was visualized using 3,3-diaminobenzidine (DAB, catalog no: TA-125-HD, Thermo Scientific, Waltham, MA, USA) as the chromogen, producing a brown-colored reaction product. Sections were subsequently counterstained with hematoxylin, dehydrated, cleared, and mounted. The stained slides were examined by two independent observers blinded to the experimental groups. NF-κB and HMGB1 expression were evaluated semi-quantitatively based on staining intensity and distribution patterns, considering both cytoplasmic and nuclear localization [50].

Staining intensity was scored as follows:

0 = no immunoreactivity;

1 = mild immunoreactivity;

2 = moderate immunoreactivity;

3 = strong immunoreactivity.

Immunohistochemical evaluations were performed using a light microscope (Imager A2, Carl Zeiss, Oberkochen, Germany) at ×200 magnification. For each sample, at least five randomly selected microscopic fields were examined, and the final score was determined based on the median value of the evaluated fields.

### 4.10. Bioinformatics Analysis

To identify potential molecular pathways associated with the experimentally observed reduction in HMGB1 and NF-κB expression following taxifolin treatment, an in silico pathway enrichment analysis was performed. First, a protein–protein interaction (PPI) network centered on HMGB1 and NF-κB was constructed using the STRING database (accessed date: 30 January 2026). The network included 50 additional interacting proteins and was subsequently visualized using Cytoscape software (version 3.10.4). The proteins identified in the PPI network were then subjected to pathway enrichment analysis using the Enrichr platform with the Reactome Pathways database (accessed date: 30 January 2026) employed as the reference dataset to identify potentially associated biological pathways [51,52]. The resulting pathways were ranked according to ascending *p*-values, and the top ten most significantly enriched pathways were reported. A *p*-value < 0.05 was considered statistically significant.

### 4.11. Molecular Docking

The three-dimensional crystal structures of TLR4 (PDB ID: 3FXI) and NF-κB p65 (PDB ID: 1NFI) were retrieved from the Protein Data Bank (PDB) [53]. Protein structures were prepared using AutoDockTools (version 1.5.7; The Scripps Research Institute, La Jolla, CA, USA) [54], where water molecules were removed and polar hydrogens and Kollman charges were added. The molecular structure of taxifolin was obtained from the PubChem database (National Center for Biotechnology Information, Bethesda, MD, USA) and converted to PDBQT format using Open Babel (version 3.1.1; Open Babel Development Team). Molecular docking simulations were performed using AutoDock Vina (version 1.2.5; The Scripps Research Institute, La Jolla, CA, USA), which is an open-source docking software (accessed date: 12 February 2026) [55]. Grid boxes were defined to cover the active binding regions of each protein, and the exhaustiveness parameter was set to 8.

### 4.12. Statistical Analysis

Statistical analyses were performed using IBM SPSS Statistics version 20.0 (SPSS Inc., Chicago, IL, USA). Normality of data distribution was assessed using the Shapiro–Wilk test. Biochemical data were presented as mean ± standard deviation, whereas histopathological and immunohistochemical scores were expressed as median with interquartile range (IQR). Differences among groups were analyzed using one-way analysis of variance (ANOVA) followed by the Tukey post hoc test for normally distributed variables. For non-parametric data, the Kruskal–Wallis test followed by Dunn–Bonferroni post hoc correction was applied. The relationship between NF-κB and HMGB1 expression scores was evaluated using Spearman correlation analysis. A *p*-value < 0.05 was considered statistically significant. Sample size estimation was performed using G*Power software (version 3.1) [56]. Based on an effect size of 0.70, α = 0.05, and statistical power (1 − β) = 0.80, the minimum required total sample size was calculated as 28 animals.

## 5. Conclusions

The present study demonstrates that taxifolin exerts significant protective effects against hepatic ischemia–reperfusion-induced remote lung injury by attenuating oxidative stress and inflammatory responses. In particular, taxifolin markedly reduced HMGB1 and NF-κB activation, suggesting that modulation of this inflammatory pathway may represent a key mechanism underlying its protective action. These findings highlight the potential therapeutic value of taxifolin in preventing remote organ injury associated with hepatic ischemia–reperfusion.

## Figures and Tables

**Table 1:** Mean ± standard deviations of serum biochemical values (MDA, IL-6, TAS, TOS) of all groups.

Groups	MDA (pg/mL)	IL-6 (pg/mL)	TAS (mmol Trolox Equivalent/L)	TOS (μmol H_2_O_2_ Equivalent/L)
Control	391.81 ± 124.57 *	30.59 ± 3.01 *	1.56 ± 0.47	1064.90 ± 32.47 *
Taxifolin	355.89 ± 245.85 *	28.47 ± 3.33 *	2.57 ± 0.31	1069.91 ± 50.52 *
Hepatic I/R	3308.15 ± 2319.50	40.33 ± 5.99	1.48 ± 0.48	1357.35 ± 64.98
Taxifolin I/R	560.90 ± 493.85 *	28.39 ± 4.11 *	2.04 ± 0.33	1129.26 ± 61.13 *

Data are presented as mean ± standard deviation. Statistical comparisons among groups were performed using one-way ANOVA followed by Tukey’s post hoc test. I/R; Ischemia/reperfusion, MDA; Malondialdehyde, IL-6; Interleukin-6, TAS; Total Antioxidant Status, TOS; Total Oxidant Status. * vs. hepatic I/R *p* < 0.05.

**Table 2:** Wet/Dry Lung Weight Ratio Across Experimental Groups.

Group	Wet Lung Weight (g)	Dry Lung Weight (g)	Wet/Dry Ratio	*p*-Value
Control	1.21 ± 0.09	0.27 ± 0.02	4.48 ± 0.21	—
Taxifolin	1.18 ± 0.11	0.27 ± 0.02	4.37 ± 0.24	0.71 vs. control
Hepatic I/R	1.63 ± 0.14	0.28 ± 0.03	5.82 ± 0.35	<0.001 vs. control/taxifolin
Taxifolin + I/R	1.36 ± 0.12	0.28 ± 0.02	4.89 ± 0.27	0.002 vs. hepatic I/R; 0.018 vs. control

Data are presented as mean ± standard deviation. Statistical comparisons were performed using one-way ANOVA followed by Tukey’s post hoc test.

**Table 3:** Lung Histopathological Injury Scores (Median [IQR]).

Parameter	Control (*n* = 7)	Taxifolin (*n* = 7)	Hepatic I/R (*n* = 7)	Taxifolin + I/R (*n* = 7)	*p*-Value †
Alveolar edema	0 (0–0)	0 (0–1)	3 (3–4) *	1 (1–2) #	<0.001
Alveolar hemorrhage	0 (0–0)	0 (0–0)	2 (2–3) *	0 (0–1) #	<0.001
Leukocyte infiltration	0 (0–0)	1 (0–1)	3 (3–4) *	2 (1–2) #	<0.001
Septal thickening	0 (0–0)	1 (0–1)	3 (3–4) *	2 (1–2) #	<0.001
Total lung injury score	0 (0–0)	2 (1–3)	11 (10–13) *	5 (4–7) #	<0.001

† Kruskal–Wallis test was applied. * *p* < 0.05 vs. control and taxifolin groups; # *p* < 0.05 vs. hepatic I/R group (Dunn–Bonferroni post hoc test).

**Table 4:** Combined Immunohistochemical Expression Scores of NF-κB and HMGB1 in Lung Tissue.

Marker	Control (*n* = 7)	Taxifolin (*n* = 7)	Hepatic I/R (*n* = 7)	Taxifolin + I/R (*n* = 7)	*p*-Value †
NF-κB expression score	0 (0–1)	1 (0–1)	3 (2–3) *	1 (1–2) #	<0.001
HMGB1 expression score	1 (0–1)	1 (0–1)	3 (2–3) *	1 (1–2) #	<0.001

† Kruskal–Wallis test was applied. * *p* < 0.05 vs. control and taxifolin groups; # *p* < 0.05 vs. hepatic I/R group (Dunn–Bonferroni post hoc test). Data was presented median (IQR).

**Table 5:** Correlation between NF-κB and HMGB1 Expression Scores.

Variables	Spearman r	*p*-Value
NF-κB vs. HMGB1	0.82	<0.001

## Data Availability

The datasets generated and/or analyzed during the current study are available from the corresponding author upon reasonable request.

## Abbreviations

ALI	Acute Lung Injury
ARRIVE	Animal Research: Reporting of In Vivo Experiments
DAB	Diaminobenzidine
DAMP	Damage-associated molecular pattern
DNA	Deoxyribonucleic Acid
EDTA	Ethylenediaminetetraacetic Acid
ELISA	Enzyme-Linked Immunosorbent Assay
GO	Gene Ontology
H&E	Hematoxylin—Eosin
HMGB1	High Mobility Group Box 1
I/R	Ischemia–Reperfusion
IL-6	Interleukin-6
KEGG	Kyoto Encyclopedia of Genes and Genomes
LPS	Lipopolysaccharide
MD2	Myeloid differentiation protein 2
MDA	Malondialdehyde
NF-κB	Nuclear Factor-kappa B
PBS	Phosphate-Buffered Saline
PDB	Protein Data Bank
PPI	Protein–Protein Interaction
ROS	Reactive Oxygen Species
TAS	Total Antioxidant Status
TBA	Thiobarbituric Acid
TLR4	Toll-Like Receptor 4
TOS	Total Oxidant Status
